# A Rare Case of Metastasis of Gastric Adenocarcinoma to the Leptomeninges

**DOI:** 10.7759/cureus.37120

**Published:** 2023-04-04

**Authors:** Krishna Doshi, Edward James

**Affiliations:** 1 Internal Medicine, Advocate Lutheran General Hospital, Park Ridge, USA; 2 Oncology, Advocate Lutheran General Hospital, Park Ridge, USA

**Keywords:** neuroimaging, malignancy, signet ring adenocarcinoma, leptomeningeal carcinomatosis, gastric adenocarcinoma

## Abstract

Leptomeningeal carcinomatosis (LMC) is defined as the diffuse infiltration of malignant cells throughout the pia and arachnoid membrane. LMC is commonly observed in patients with leukemia, lymphoma, and breast and lung cancer. The prevalence of LMC spread in patients with primary gastric malignancy is very rare. Due to its devastating neurological complications and high mortality, it is difficult to assess the associated clinical features, treatment outcomes, and prognostic factors. Current treatment options include intra-thecal chemotherapy, radiotherapy, and supportive care with a median survival of three to four months. LMC is a rare manifestation of gastric cancer and is an extremely fatal disease. Therefore, it is difficult to distinguish LMC from other neurological etiologies. We present a unique case of an individual who presented with headaches and was found to have LMC.

## Introduction

Gastric cancer (GC) is the fourth leading cause of death worldwide, with median survival rates being 12 months in patients with advanced gastric malignancy [1]. Leptomeningeal carcinomatosis (LMC) usually occurs in patients with leukemia, lymphoma, breast, melanoma, and lung cancer [2]. LMC can present in patients with multifocal neurological deficits such as infiltration of cranial and spinal nerve roots, obstructive hydrocephalus, or a combination of these. It has been reported that the incidence of gastric metastasis to leptomeninges is rare and about 0.06% of all GC cases [3]. Several studies have hypothesized that the majority of LMC patients with advanced gastric cancer have metastasis to the leptomeninges using Batson's plexus [3-6]. Due to its rare occurrence, the clinical signs and prognostic factors have not been accurately identified, and treatment options remain limited, ultimately making it a fatal disease with a median survival of three to four months, as reported by Bulut et al. [5].

Current treatment options include intra-thecal (IT) chemotherapy, radiotherapy, and supportive care in patients with poor performance status. Methotrexate is the common choice for IT chemotherapy, although cytarabine and thiotepa have been used in combination with hydrocortisone [3]. Radiotherapy has been introduced in patients with high tumor burden. Due to its fatal nature, the effectiveness of current therapies is difficult to assess in patients. There are very few reports on LMC arising from gastric malignancy as primary. Therefore, we present a unique case of an individual in remission developing headaches and later found to have LMC.

## Case presentation

A 50-year-old female patient of Asian descent with a past medical history significant for gastric cancer presented to the hospital in June 2022 for an ongoing headache that was present for the prior two weeks.

Her previous oncologic history is as follows. She was diagnosed with gastric cancer involving the antrum in February 2016 after complaints of worsening dyspepsia. Initial endoscopic ultrasound/ upper endoscopy showed a large gastric mass, T4aN+. Biopsy revealed poorly differentiated adenocarcinoma with focal signet ring cell differentiation. The patient then underwent neoadjuvant leucovorin calcium (folinic acid), fluorouracil, and oxaliplatin (FOLFOX) therapy for four cycles, followed by chemoradiation with capecitabine and subtotal gastrectomy with D2 extra-regional lymph node dissection at an outside institution.

Postoperative pathology showed pT3pN3cM0. She was in clinical remission until January 2022, when magnetic resonance imaging (MRI) pelvis showed a 14.5 x 7.7 x 9.6 cm mass involving the left ovary, concerning of neoplasm. She then underwent a total abdominal hysterectomy and bilateral salpingo-oophorectomy (TAH-BSO) at an outside institution with pathology demonstrating metastatic adenocarcinoma with signet ring morphology consistent with her prior diagnosis of gastric cancer. The tumor was microsatellite stable, HER2 negative, PD-L1 CPS <1; next-generation sequencing showed MET amplification and ARID1A and TERT mutations. Liquid biopsy was negative for any alterations. No additional chemotherapy was offered per a review of records from the outside institution.

Computerized tomography (CT) imaging with contrast done in March 2022 showed no evidence of additional metastatic disease. However, CT imaging in June 2022 demonstrated new mediastinal, hilar, retroperitoneal, and pelvic lymphadenopathy and non-specific nodularity involving the vaginal cuff, which was concerning for metastatic recurrence. 

She started having headaches in June 2022, which was initially followed as an outpatient. Due to worsening headaches with intermittent nausea and the development of left eye ptosis, the patient presented to the emergency department (ED). An MRI in the ED revealed dural enhancement with concern for subacute subarachnoid hemorrhage. Subsequently, CT angiography head and neck and CT venography brain were obtained, which did not demonstrate vascular abnormalities.

**Figure 1:**
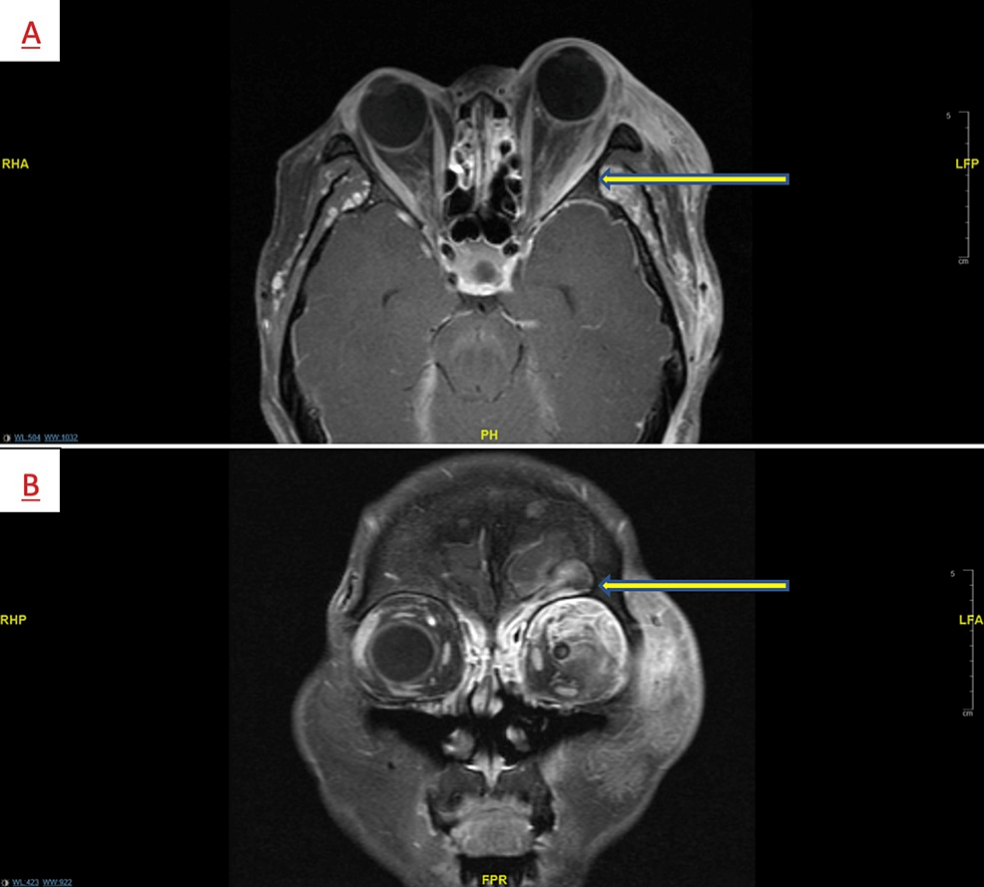
MRI orbits with and without contrast 1A and 1B: There are diffusely thickened superior and lateral rectus muscles of the left orbit. There is an abnormal enhancement along the adjacent roof and lateral walls of the left orbit. However, there is proptosis of the left orbital globe. The left optic nerve is normal. There is preseptal periorbital soft tissue swelling. There is diffusely thickened enhancing dura surrounding the visible frontal lobes bilaterally. The findings are most suspicious for orbital cellulitis in addition to preseptal cellulitis on the left side. A less likely possibility would include metastatic infiltration.

She was admitted to the neurocritical care unit for further evaluation. An MRI orbit (Figure 1) was obtained, which showed concern for orbital cellulitis in addition to preseptal cellulitis with the possibility of metastasis. A large volume lumbar puncture was performed, which was notable for high protein and opening pressure of 32cm H20. Rapid meningitis panel, cytology x2, mayo encephalopathy panel, and paraneoplastic panel were negative. During this time, the patient continued to express concerns about her headache which was waxing and waning. The left eye ptosis/proptosis had worsened. However, the patient remained afebrile throughout her stay. She subsequently experienced a pulseless electrical activity (PEA) cardiac arrest. Return of spontaneous circulation was achieved after three minutes of cardio-pulmonary resuscitation and one dose of epinephrine. The patient was intubated for acute respiratory failure. CT pulmonary embolism was negative. Due to the absence of brainstem reflexes, urgent CT brain was obtained, which demonstrated effacement of the basal cisterns due to brain sagging and concern for intracranial hypotension. Subsequently, the patient was empirically given mannitol and 23% saline with the return of her pupillary reflexes. Neurosurgery was notified, and an external ventricular drain was placed as intracranial pressure was noted to be in the 40s. Following this, the patient received a diagnostic cerebral angiogram, which showed no concerns for cerebral vasculitis. Deep and superficial venous system was patent, and no signs of an aneurysm or other vascular malformations were noted. Repeat CT brain two days later showed left thalamic ischemic stroke and stable posterior fossa. Empiric steroids were started, and an electroencephalogram (EEG) was initiated, which showed frequent L sharps and no seizures. Once stable, the patient was taken for a dural biopsy which was consistent with metastatic poorly differentiated adenocarcinoma involving the dural lymphovascular spaces, not amenable for treatment. The patient was subsequently pronounced to be brain-dead after appropriate clinical exams were completed.

## Discussion

Leptomeningeal carcinomatosis (LMC) is defined as a diffuse infiltration of malignant cells within the pia mater and the arachnoid mater. Per literature, there are several possible avenues through which tumor cells migrate to the leptomeninges, such as arterial circulation, retrograde flow through Batson's plexus, and perineal or perivascular spaces [3-7]. LMC usually occurs in three to eight percent of all cancer patients leading to increased neurological morbidity and mortality [3]. Breast cancer, malignant melanoma, lung cancer, lymphomas, and leukemia are the most common malignancies that metastasize to the leptomeninges [5]. As reported by Kim et al., the incidence of LMC arising from gastric cancer as the primary malignancy is 0.16-0.69% [6-7]. LMC from gastric cancer is extremely rare, and the most common histopathologic type of gastric cancer associated with LMC is signet ring adenocarcinoma [5-6].

Leptomeningeal carcinomatosis usually presents in patients diagnosed with advanced stages of malignancies with extensive disease involvement in the liver, lungs, and bones [8]. Common symptoms associated with LMC can be non-specific such as headache, nausea or vomiting, dizziness, and altered mental status, making it difficult to recognize [3]. Le et al. illustrated that the overall median survival for patients diagnosed with LMC is approximately four to six weeks if untreated and two to four months if treatment is initiated [9,10]. However, our patient did not have extensive disease spread at the time of her presentation. Current literature illustrates that the time lapse between the initial diagnosis of gastric cancer and LMC is approximately 12 months [8]. This was not the case in our patient, who showed signs many years after her initial diagnosis.

Definitive diagnosis of LMC requires radiographic imaging. However, imaging might not always reveal LMC. Current literature demonstrates that the sensitivity of MRI in the diagnosis of LMC is between 65% to 75% [5]. Another modality is cerebrospinal fluid (CSF) sampling, which has been reported to have a sensitivity of 54% and, with repeat samplings, up to 91% [9]. Our patient's MRI demonstrated inflammation and was presumed to be cellulitis due to the history of the patient. CSF sampling could not be performed as our patient became clinically unstable for invasive intervention, therefore, making it difficult to diagnose her LMC.

The current standard of care for patients diagnosed with LMC is treatment with methotrexate for intra-thecal (IT) administration. A combination of IT chemotherapy with methotrexate arabinoside and hydrocortisone has been reported to be more effective than methotrexate alone [6,11]. Treatment options for patients with gastric cancer and LMC are systemic chemotherapy using the FLOT protocol (docetaxel, oxaliplatin, leucovorin, and 5-fluorouracil) have shown promising outcomes in managing patients with advanced gastric cancer [12].

Moreover, recent developments made in the field by the RAINBOW trial have shown that anti-vascular endothelial growth factor (VEGFR) therapies such as ramucirumab are useful in achieving prolonged overall survival [13]. More recent innovations include testing human epidermal growth factor receptor 2 (HER2) overexpression and its targeted treatment with trastuzumab, which has shown results on the control of the disease progression when administered intrathecally [14]. Our patient was HER-2 negative, microsatellite stable gastric cancer with a low combined positive score (CPS) score, which precluded treatment options targeting HER-2 and immunotherapy options. Next-generation sequencing was performed at an outside institution, which did not show any actionable alterations.

Overall, LMC is an extremely rare manifestation of gastric malignancy and a very fatal disease. As appropriate treatment may increase the overall median survival of the patient, the recognition of LMC early is essential for prolonging length and quality of life.

## Conclusions

In conclusion, we described a case of leptomeningeal carcinomatosis with gastric malignancy as primary. Due to its catastrophic outcomes and poor prognosis, it is essential that clinicians keep this on the differential when approaching patients with primary gastric malignancy who present with non-specific neurological symptoms.
